# Effects of coal's initial macro-cracks on rockburst tendency of rock–coal composite samples

**DOI:** 10.1098/rsos.181795

**Published:** 2019-11-06

**Authors:** Shaojie Chen, Dawei Yin, Huimin Liu, Bing Chen, Ning Jiang

**Affiliations:** 1 State Key Laboratory of Mine Disaster Prevention and Control, Shandong University of Science and Technology, Qingdao 266590, People's Republic of China; 2 Shandong Key Laboratory of Civil Engineering Disaster Prevention and Mitigation, Shandong University of Science and Technology, Qingdao 266590, People's Republic of China; 3 School of Earth Sciences and Engineering, Shandong University of Science and Technology, Qingdao 266590, People's Republic of China

**Keywords:** coal's initial macro-crack, rock–coal composite sample, rockburst tendency, elastic energy attenuation index, uniaxial compressive strength

## Abstract

In the present study, uniaxial compression tests were conducted on sandstone–coal composite samples to investigate the effects of original macro-cracks in coal on the rockburst tendency. First, the energy dissipation theory was used to derive the elastic energy attenuation index of composite samples during uniaxial loading. Then, based on the test results obtained, the rockburst tendency of composite samples was evaluated and analysed using the uniaxial compressive strength and elastic energy attenuation index. The results show that the original macro-cracks in coal deteriorated the rockburst tendency of composite samples. The original horizontal cracks had the lowest effect on the rockburst tendency, whereas the vertical penetrating cracks through the coal centre (parallel to the loading direction) displayed the greatest effect. The mechanism by which these macro-cracks weakened the rockburst tendency involved two steps: (i) changing the physical properties and energy accumulation conditions of composite samples and (ii) increasing the energy dissipation of composite samples during uniaxial loading. These aspects are important to understand the rockburst hazards induced by the structural instability and failure of the composite system of coal seam and roof rock during deep coal mining.

## 1. Introduction

**Figure 1.**
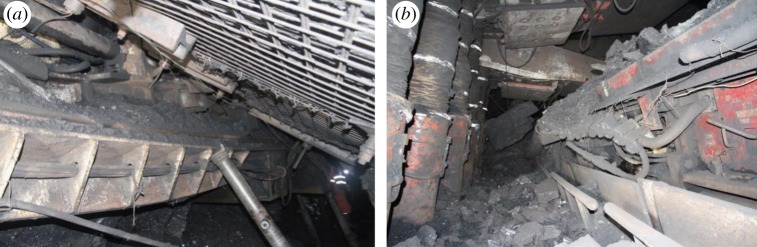
The photographs of rockburst damage in roadways and working face [9]. (*a*) H frame of belt conveyor was ejected and connected with roof. (*b*) Shearer was seriously tilted and damaged.

With the shallow coal resources in China rapidly getting exhausted, coal mining has transferred to the deeper parts. For instance, the mining depth of the Huafeng coal mine located in eastern China has reached −1300 m (below the surface). However, deep mining is considerably hazardous as the complex geological conditions and high-stress environment at such depths induce frequent occurrences of rockburst hazards, resulting in huge economic losses and casualties [1–9], as shown in figure 1. Numerous studies have been conducted to find the mechanism and predict rockburst disasters in coal mines. Preliminary investigations have indicated potential connections between the occurrence of rockburst hazards and rockburst tendency of coal or rock samples [10–15]. The rockburst tendency of coal or rock samples is used as the primary predictive index to assess the probability of rockburst disasters. The evaluation indexes of rockburst tendency are obtained from laboratory tests or numerical simulations of coal or rock samples [16]; these indexes include dynamic failure time (*D*_T_), elastic energy index (*W*_ET_), bursting energy index (*K*_E_) and uniaxial compressive strength (UCS, denoted by *R*_C_). *D*_T_ refers to the time from the ultimate compressive strength to complete failure of coal or rock samples under uniaxial loading. *W*_ET_ and *K*_E_ are defined as per equation (1.1) [16]; currently, the brittleness index and elastic energy attenuation index are used for evaluating the rockburst tendency.

1.1 $$\begin{array}{cc} & W_{\mathrm{ET}}=\frac{Q_{\mathrm{SE}}}{Q_{\mathrm{SP}}}\\ \text{and}\; & K_{E}=\frac{F_{s}}{F_{x}} \end{array}\}$$

where *Q*_SE_ and *Q*_SP_ are the elastic strain energy and plastic strain energy of samples under uniaxial unloading, respectively, when the loading stress reaches a certain value (before failure); *F*_s_ is the accumulative deformation energy of samples before peak stress under uniaxial loading; and *F*_x_ is releasable deformation energy of samples after peak stress under uniaxial loading.

The evaluation indexes for assessing rockburst tendency for pure coal or rock samples for a shallow coal seam can predict well the occurrence of rockburst events. However, a large number of engineering practices show that rockburst disasters can be induced by the structural instability and failure of the composite system of coal seam and roof rock layer under deep coal mining influences [4–6,17,18]. Lu *et al.* found that rockburst disasters can be induced by the collapse of a hard roof rock [9]. Similarly, Qi *et al.* proposed that the sliding of the roof rock was the primary factor inducing rockburst disasters in an inclined seam mining [19]. Therefore, the rockburst tendency of composite samples of coal and rock should be used as the prediction index for assessing the probability of rockburst disasters during deep coal mining. Several experiments and numerical simulations have been performed to evaluate the rockburst tendency of composite samples of rock and coal. The results of these studies have led to further investigations into the effects of the height ratio of rock to coal, strength and homogeneity of rock and interface angles between rock and coal on the rockburst tendency of rock–coal, coal–rock and rock–coal–rock composite samples [20–23]. These studies reported that with an increase in the height ratio of rock to coal, and strength and homogeneity of rock, the rockburst tendency of composite samples increased. Moreover, compared with the pure coal or rock samples, the rockburst tendency of composite samples was relatively large.

Unlike the top and bottom rock layers, the coal seam generally contains several original or pre-existing cracks or joints, which affect the occurrence of rockburst disasters. Hao *et al.* analysed the effects of the bedding plane angle on the rockburst tendency of pure coal samples [24]. The rockburst tendency of pure coal samples increased with an increase in the bedding plane angle, as measured from the horizontal direction. Similarly, the initial cracks or joints in coal affect the rockburst tendency of rock–coal composite samples. However, the literature on this aspect is scarce. Previous investigations on the rockburst tendency of composite samples have focused more on intact samples and neglected the effects of the original defects in the coal.

In the present study, we derived the elastic energy attenuation index of composite samples based on the energy dissipation theory of rock–coal composite samples. Subsequently, the uniaxial compression tests were conducted on sandstone–coal composite samples having pre-existing macro-cracks in coal. *R*_C_ and the elastic energy attenuation index were used as evaluation indexes, and the effects of original macro-cracks on the rockburst tendency were analysed and discussed.

## 2. Elastic energy attenuation index of rock–coal composite samples

Coal mining results in a balanced system that consists of energy consumption and external energy input in the composite system of coal seam and roof rock layer. Rockburst disasters are a dynamic phenomenon characterized by a sudden release of elastic energy stored in the composite system during coal mining. Therefore, the elastic energy attenuation index can be used to evaluate the rockburst tendency of composite samples.

The elastic strain energy (*U*) of a rock–coal composite sample under uniaxial loading is given by [25–27]

2.1 $$U=\frac{1}{2}\;\sigma \varepsilon_{e}=\frac{\sigma^{2}}{2E},$$

where *E* is the elastic modulus of the composite sample, *ε*_e_ is the elastic strain of the composite sample under uniaxial loading and *σ* is the axial stress. The axial stress is given by

2.2 $$\sigma =E\varepsilon \exp\left[-{\left(\frac{\varepsilon }{\varepsilon_{0}}\right)}^{m}\right],$$

where *ε* is the total strain of the composite sample under uniaxial loading, *ε*_0_ is the distribution scale of the Weibull distribution and *m* is the morphological parameter characterized in the strain form [26].

*U* is determined by combining equations (2.1) and (2.2) as follows:

2.3 $$U=\frac{1}{2}\sigma \varepsilon_{e}=\frac{E\varepsilon^{2}\exp[-2{\left(\varepsilon /\varepsilon_{0}\right)}^{m}]}{2}.$$

Therefore, the elastic energy attenuation index (*η*) is

2.4 $$\eta =\frac{\partial U}{\partial \varepsilon }=\frac{\sigma }{E}\frac{\partial \sigma }{\partial \varepsilon }=E\varepsilon \left[1-m{\left(\frac{\varepsilon }{\varepsilon_{0}}\right)}^{m}\right]\exp\left[-2{\left(\frac{\varepsilon }{\varepsilon_{0}}\right)}^{m}\right].$$

As *m* is 1, equation (2.4) can be rewritten as [21]

2.5 $$\eta =\frac{\partial U}{\partial \varepsilon }=\frac{\sigma }{E}\frac{\partial \sigma }{\partial \varepsilon }=E\varepsilon \left[1-\frac{\varepsilon }{\varepsilon_{0}}\right]\exp\left[-2\frac{\varepsilon }{\varepsilon_{0}}\right].$$

According to equation (2.5), *η* is positively correlated with *E*. Specifically, when *E* is larger, *η* is greater and the release of elastic energy stored in the composite samples is more severe. Consequently, the rockburst tendency of composite samples is high.

The value of *E* is determined by the elastic moduli and heights of rock and coal in the composite sample, which is

2.6 $$\begin{array}{r} E=\frac{E_{r}E_{c}L}{E_{c}H+E_{r}h} \end{array},$$

where *E*_r_ and *H* are the elastic modulus and height of the rock in the composite sample, respectively; *E*_c_ and *h* are the elastic modulus and height of coal, respectively. *L* is the height of the composite sample. The rock–coal composite sample is a standard sample (*ϕ*50 mm × 100 mm). Therefore, *L* is 100 mm and equal to the sum of *H* and *h*.

Equation (2.6) can be changed as

2.7 $$\frac{1}{E}=\frac{1}{E_{r}}+\left(\frac{1}{E_{c}}-\frac{1}{E_{r}}\right)\frac{1}{1+(H/h)}.$$

In equation (2.7), under the same conditions, *E* is positively correlated to the height ratio of rock to coal in the composite sample. This means that when the height ratio of rock to coal is higher, *E* and *η* are greater. Moreover, the rockburst tendency of composite samples increases with the height ratio of rock to coal. This finding is consistent with those obtained in previous experimental and simulation studies on the effects of the height ratio of rock to coal on the rockburst tendency of composite samples [20].

Compared with the roof and floor rock layers, the coal seams generally contain several original or pre-existing cracks or joints. According to the previous investigations, the defects (especially penetrative and surface macro-cracks) affect the elastic modulus of pure coal or rock samples [27–31]. This is reflected in equation (2.6): the original macro-cracks of coal affect *E*_c_, consequently influencing *E*. Finally, *η* is affected by the pre-existing macro-cracks of coal, which also affect the rockburst tendency of composite samples. These effects are manifested in different ways owing to various characteristics of the original macro-cracks of coal, which were analysed based on the uniaxial test results of the sandstone–coal composite samples with original macro-cracks in coal.

## 3. Material and methods

### 3.1. Sample preparation

**Figure 2.**
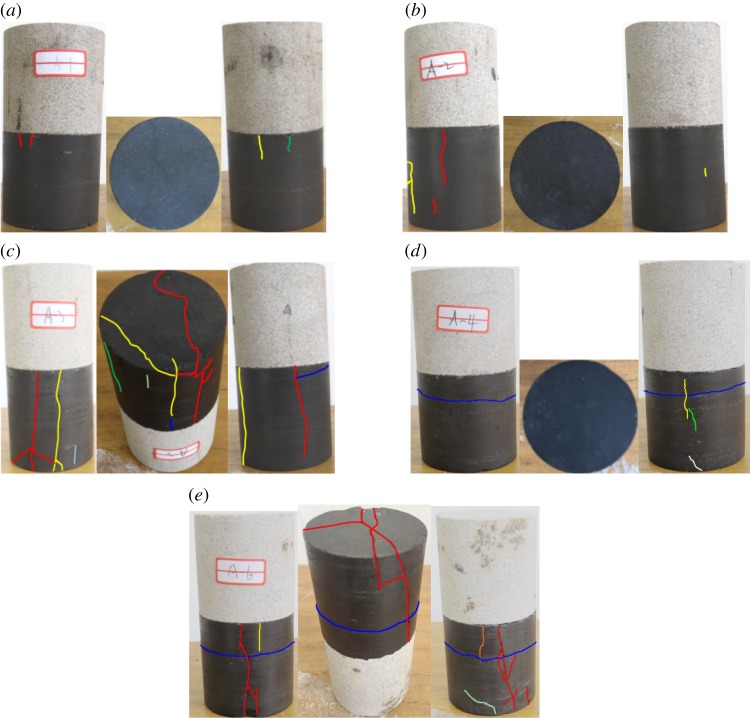
Sandstone–coal composite samples with initial macro-cracks in coal. (*a*) A-1 composite sample. (*b*) A-2 composite sample. (*c*) A-3 composite sample. (*d*) A-4 composite sample. (*e*) A-5 composite sample.

Coal and sandstone blocks were selected from the Daizhuang coal mine in the Shandong Province of China. Sandstone is the immediate roof. These blocks were densely drilled into coal and sandstone samples. While investigating its mechanical properties, the composite system of coal seam and roof rock layer was simplified as rock–coal composite samples with a bonded or frictional interface in the previous laboratory tests or numerical simulations [5,6,9,15,16,19–22]. According to the previous investigations, the intact sandstone samples and coal samples with typical original macro-cracks were selected and bonded into standard composite samples (*ϕ*50 mm × 100 mm; figure 2) using superglue. The height ratio of sandstone to coal was 1 : 1 in the composite samples.

Distributions of the initial macro-cracks in coal are presented in table 1. All coal in the composite samples was cracked to some extent. However, a coal sample with a minimum number of critical cracks and lowest persistence was considered to be an intact coal (no-crack coal) reference. The A-1 composite sample displayed the lowest crack intensity, persistence and total number; thus, it was selected to represent the intact sandstone–coal composite sample.

**Table 1.** Distributions of the initial macro-cracks in coal.

sample no.	main distribution of the initial macro-cracks in coal
A-1	four small vertical surface cracks adjacent to the sandstone–coal interface (minimum persistence) (intact composite sample)
A-2	two large vertical surface cracks: one adjacent to the sandstone–coal interface and the second in the middle and lower parts of the coal
A-3	two vertical penetrative side-cracks through the coal near the lateral boundary
A-4	one horizontal penetrative crack and some surface cracks
A-5	one main vertical penetrating crack through the central body of the coal and the other cracks being partially penetrative through the coal body. One horizontal penetrative crack in the lower part of the coal near the sandstone–coal interface

### 3.2. Test system

Uniaxial compression tests in sandstone–coal composite samples with original macro-cracks in coal were conducted using an AG-X250 servo-controlled testing system. A double screw loading structure was used for working flexibility, which allowed the testing system to execute conventional compression, tensile or any other mechanical tests, as required. The maximum testing load can reach up to 250 kN. A displacement loading method at a loading rate of 0.0005 mm s^−1^ was adopted in these tests.

## 4. Results

**Figure 3.**
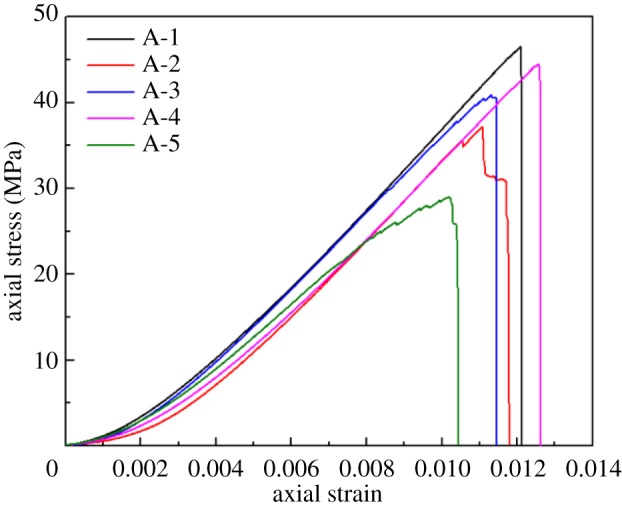
Uniaxial compression stress–strain curves of composite samples with original macro-cracks in coal.

Figure 3 shows the uniaxial compression stress–strain curves of five sandstone–coal composite samples. Uniaxial test results are listed in table 2, including the UCS, peak strain and *E*.

**Table 2.** Uniaxial compression test results of composite samples.

no.	UCS (MPa)	peak strain	*E* (GPa) (at 40–60% of peak stress)
A-1	46.55	0.01209	4.505
A-2	31.79	0.01108	4.267
A-3	40.88	0.01131	4.468
A-4	44.52	0.01256	4.370
A-5	28.99	0.01021	3.843

**Figure 4.**
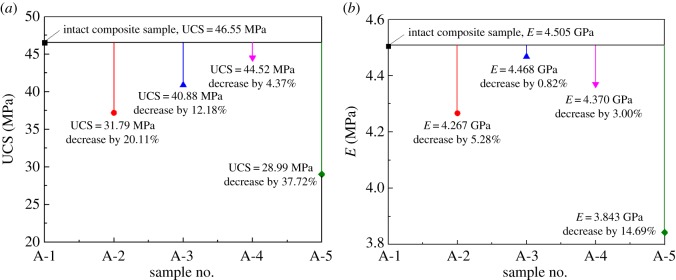
Comparisons of UCS and *E* for composite samples. (*a*) UCS comparison. (*b*) *E* comparison.

As seen in figure 2, the trends in the stress–strain curves of composite samples with pre-existing macro-cracks in coal were basically consistent with those of the intact composite sample (A-1 composite sample). However, the presence of pre-existing macro-cracks in coal led to wide variations in UCS and *E* of composite samples. UCS and *E* of composite samples are compared in figure 4*a*,*b*, respectively.

Previous studies have shown that the rockburst tendency of pure coal or rock samples increased with an increase in UCS [20,21]. Correspondingly, with an increase in UCS, the rockburst tendency of composite samples increased. Meanwhile, *E* exhibited a positive relationship with *η*. Specifically, the rockburst tendency increased with *E.* The UCS of the intact composite sample was 46.55 MPa, and the corresponding value of *E* was 4.505 GPa. As seen in figure 4, the values of UCS and *E* of composite samples with the original macro-cracks in coal were smaller than those of the intact composite sample. It is proved that the initial macro-cracks of coal deteriorated the rockburst tendency of composite samples.

According to the test results, the negative effects were

(1) When the initial macro-crack in coal was mainly a horizontal penetrative crack (A-4 composite sample), the values of UCS and *E* were recorded to be 44.52 MPa and 4.370 GPa, respectively. These values were just slightly less than the maximum reference value, suggesting that a horizontal penetrative crack had a small negative impact on the rockburst tendency.
(2) When the initial macro-cracks in coal were mainly vertical penetrating cracks through the coal centre (A-5 composite sample), UCS decreased to a minimum value of 28.99 MP, a 37.72% strength reduction compared with the intact composite sample. The corresponding *E* was the lowest (3.834 GPa) in this test. The vertical penetrating cracks through the coal centre had the largest negative impact on the rockburst tendency.
(3) Compared with the intact composite sample, the values of UCS and *E* in the A-2 composite sample decreased by 20.11% and 5.28%, respectively. These values show that the vertical surface cracks in coal had a large negative impact on the rockburst tendency.
(4) Vertical penetrating cracks close to the lateral surface boundary (A-3 composite sample) had a great effect on UCS but a small effect on *E.* Compared with the intact composite sample, UCS and *E* decreased by 12.18% and 0.82%, respectively. From this point of view, the initial macro-cracks in coal had a relatively large deterioration effect on the rockburst tendency. By contrast, the initial macro-cracks in coal exhibited a relatively low deterioration effect on the rockburst tendency in terms of *E*. Moreover, the deterioration effect was smaller than that in the A-4 composite sample. These findings could be attributed to the definition of *E* in the present study. Overall, the vertical penetrating cracks close to the lateral surface boundary had a negative impact on the rockburst tendency.

The above analyses revealed that the horizontal penetrative crack had the smallest negative impact on the rockburst tendency of composite samples. By contrast, the vertical penetrating cracks through the coal centre had the largest negative impact on the rockburst tendency. These conclusions were consistent with the effects of the bedding plane angle on the rockburst tendency of pure coal samples [24], verifying the accuracy of the test results. During deep coal mining, close attention must be paid to the areas of coal seam with horizontal penetrative cracks. In these areas, some measures should be adopted to decrease the occurrence probability of rockburst disasters caused by the instability and failure of the composite system of coal seam and roof rock. These measures include coal seam infusion, advanced bore decompression in the coal seam and cutting a pressure-relief slot in coal seam.

## 5. Discussions

Uniaxial loading results in a balanced system between energy consumption and external energy input in the rock–coal composite sample. The balanced state of the system is attributed to the thermodynamics laws, according to which the system is in a low energy state. Exposure to an external energy source breaks this original balance, causing the weak structures of the system to spontaneously release energy to maintain the balance. However, if the external energy input exceeds the energy consumed by the system, the surplus energy (mainly for elastic strain energy) is released in the form of mechanical energy that disintegrates the stability of the composite sample. This ultimately makes the composite sample prone to rockburst hazards.

**Figure 5.**
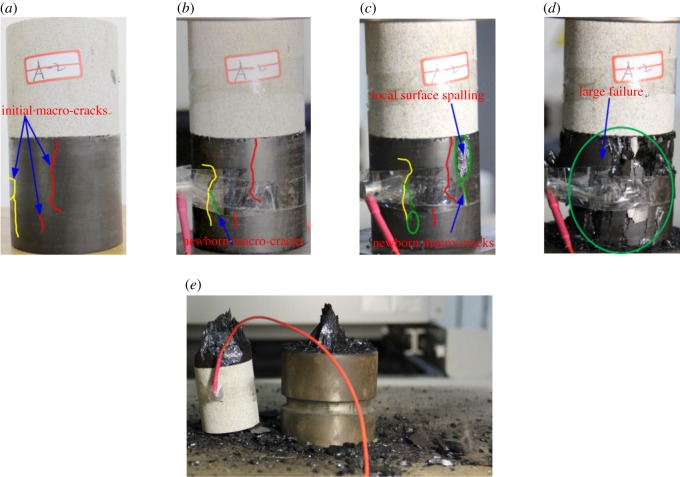
Progressive failure of the sandstone–coal composite sample with the initial macro-cracks in coal. (*a*) Initial state. (*b*) Initiation and propagation of original macro-cracks in the coal. (*c*) Local surface spalling. (*d*) Large failure. (*e*) Final failure.

For composite samples with pre-existing macro-cracks in coal, these cracks serve as weak structures in the balanced system. Before the structural instability and failure, the stress gets concentrated generally at the tips of cracks under axial stress. When the stress intensity factor (*K*_I_) at a crack tip reaches the material fracture toughness (*K*_IC_), cracks are formed and propagated further, thereby forming new cracks in the coal of composite samples; this process involves the consumption of a considerable amount of energy. However, owing to the non-homogeneity of the initial macro-cracks in the coal, these cracks may not initiate at the tips, as shown in figure 5*b*. Meanwhile, the combination of both initial and new cracks results in local structure disintegration of coal of composite samples, such as surface spalling (figure 5*c*). The low strength of coal causes the local failures to induce energy-dependent chain destruction, thereby forming a large failure area (figure 5*d*). At the same time, these reduce the bearing area of the main bearing body of coal, consequently reducing the structural strength of the whole composite sample. This is the reason for a lower UCS of composite samples with initial macro-cracks in coal when compared with that of intact composite samples. Therefore, the surplus energy is relatively low at the site of instability and failure in composite samples. In addition, according to equations (2.1) and (2.6) and study results, the pre-existing cracks weaken *E* and reduce the elastic strain energy accumulation in the composite samples. Therefore, the rockburst tendency of composite samples with the initial macro-cracks in the coal is weakened.

The above analysis revealed the negative effects of original macro-cracks on the rockburst tendency of composite samples to be mainly twofold: (1) changes in the physical properties (*E*) and energy accumulation conditions of composite samples and (2) increases in energy dissipation of the composite samples.

## 6. Conclusion

The UCS evaluation index and elastic energy attenuation index were employed to assess the effects of pre-existing macro-cracks in coal on the rockburst tendency of sandstone–coal composite samples. The original macro-cracks in the coal negatively affected the rockburst tendency of composite samples. Among these, the initial horizontal cracks displayed the lowest effect on the rockburst tendency, as evident from the decrease in the corresponding UCS and elastic modulus by 4.37% and 3.00%, respectively, compared with the intact composite sample. By contrast, the initial vertical penetrating cracks through the coal centre (parallel to the loading direction) significantly affected the rockburst tendency. The UCS and elastic modulus of the composite sample decreased by 37.72% and 14.69% compared with the intact composite sample, respectively. Based on the findings of the present study, we concluded the mechanism of weakening of rockburst tendency of composite samples based on thermodynamics laws, mainly acting in two ways: changes in the physical properties and energy accumulation conditions of the composite samples, and increases in the dissipation of the energy in the composite samples.

## Data accessibility

Data discussed in the present study were obtained from laboratory tests. The data of uniaxial test results of composite samples are described in detail in the ‘Results’ section (§4) of this manuscript.
